# PHGDH Is Upregulated at Translational Level and Implicated in Platin-Resistant in Ovarian Cancer Cells

**DOI:** 10.3389/fonc.2021.643129

**Published:** 2021-06-10

**Authors:** Fangfang Bi, Yuanyuan An, Tianshui Sun, Yue You, Qing Yang

**Affiliations:** Department of Obstetrics and Gynecology, Shengjing Hospital of China Medical University, Shenyang, China

**Keywords:** PHGDH, DDX3X, platin-resistant, ovarian cancer cells, RMRP

## Abstract

**Background:**

Platinum-based chemotherapy is the first line option for ovarian cancer. The development of resistance to such chemotherapy results in treatment failure, while the underlying mechanisms are poorly understood.

**Methods:**

Clinical samples were collected from Shengjing Hospital of China Medical University. MTT assay was used to see the proliferation and chemoresistance of ovarian cancer cells. Transwell migration and Matrigel invasion assays was used to see the invasion ability of ovarian cancer cells. In addition, polysome profiling and tissue microarray and immunohistochemical staining were also used. The statistical significance of the difference was analyzed by ANOVA and *post hoc* Dunnett’s test.

**Results:**

PHGDH is the first enzyme responsible for serine biosynthesis pathway. The current study demonstrated that PHGDH is upregulated in platin-resistant ovarian cancer cells and tissues at the protein level. Importantly, knockdown of PHGDH suppressed, while overexpression of PHGDH increased the survival upon cisplatin exposure, invasiveness and spheroid formation of ovarian cancer cells. The current study demonstrated that PHGDH translation was upregulated in platin-resistant ovarian cancer. In addition, our study provided evidence that LncRNA RMRP (RNA Component of Mitochondrial RNA Processing Endoribonuclease) was upregulated in platin-resistant ovarian cancer, which promoted enrichment of RNA binding protein DDX3X (DEAD-Box Helicase 3 X-Linked) on the PHGDH mRNA to promote its translation.

**Conclusion:**

Collectively, the current study described that PHGDH was upregulated and conferred resistance of ovarian cancer cells to cisplatin, suggesting that cisplatin resistance could be overcome by targeting PHGDH. Our study also provided evidence that differential PHGDH protein expression was defined by its translation, and RNA binding protein DDX3X and LncRNA RMRP are regulators of its translation.

## Background

Early-stage ovarian cancer is rarely detected until it progresses to late stage. Currently tumor debulking surgery combined with platinum-based chemotherapy is commonly used for ovarian cancer therapy. In many instances, however, some tumor cells are intrinsically resistant to platinum, furthermore, some initially responsive patients relapse due to acquired cisplatin resistance (1). The mechanisms underlying intrinsic or acquired platinum resistance are not completely clarified.

Alike as many cytotoxic cancer chemotherapies increase reactive oxygen species (ROS) levels, platinum compounds increase mitochondrial ROS by forming adducts on mitochondrial DNA, thereby compromising redox homeostasis (2). Not surprisingly the capacity to maintain redox homeostasis plays an important role in cisplatin-resistance (3–7). Redox homeostasis is balanced by levels between oxidants and antioxidants. Glutathione in a reduced form is the principal cellular antioxidant, which is maintained by generation of Nicotinamide Adenine Dinucleotide Phosphate (NADPH). Two shunt pathways branched from glycolysis, pentose phosphate pathway and serine synthesis pathway are responsible for intracellular NADPH generation *via* utilizing glucose intermediate metabolites (8–12). The serine biosynthetic pathway is a branching pathway diverting from glycolysis by conversion of 3-phosphoglycerate into serine. Serine is subsequently utilized as a substrate for one-carbon (folate cycle) metabolism, and biosynthesis of sphingolipids, nucleotides, and glutathione. In addition, the intracellular methionine pool and, thereby, methyl donor reactions are also supplemented by serine and glycine (13–15). Thus, serine synthesis pathway might play an important role in regulating chemoresistance of ovarian cancer cells.

Phosphoglycerate dehydrogenase (PHGDH) is the rate-limiting enzyme responsible for the serine biosynthetic pathway. It has been reported that PHGDH is upregulated in some cancers derived from distinct histology and functions as an oncogenic gene (11, 16–22). In addition to its role in catalyzing *de novo* serine synthesis, it has been reported that PHGDH promotes caner progression by production of D-2-hydroxyglutarate (D-2HG), an oncometabolite (23, 24). Genomic amplifications of PHGDH gene are observed in some breast cancers and melanomas (19, 25). PHGDH is also transcriptionally regulated by various tumor suppressors and oncogenes (26–28). Posttranslational regulation *via* proteasomal degradation is also responsible for PHGDH expression in some cancers (29). The current study identifies a novel regulatory level at translational activation responsible upregulation of platinum-resistant ovarian cancer cells. In addition, PHGDH increases resistance of ovarian cancer cells to cisplatin and promotes invasiveness and spheroid formation of ovarian cancer, making PHGDH a potential target for ovarian cancer therapy.

## Materials and Methods

### Cell Culture

Human ovarian cancer cell lines and their cisplatin resistant cohorts SKOV3 and SKOV3/DDP, A2780 and A2780/DDP were maintained in RPMI1640 containing 10% fetal bovine serum and 100 IU/ml of penicillin, 100 µg/ml of streptomycin. The cells were incubated in a humidified atmosphere at 37°C with 5% CO2.

### Sphere Formation Assay

The cells in the logarithmic growth phase were harvested and resuspended in serum-free F12 medium supplemented with 20 mg/ml Epidermal Growth Factor (EGF), 5 μg/ml Insulin and 2% B27. Cells were plated in six-well Corning Spheroid Microplates. The media were changed every 3 days, images of cells were taken under an inverted microscope and the numbers of spheroid were counted.

### Western Blotting

In order to extract total protein, cells were lysed with ice-cold RIPA lysis buffer for 30 min, then centrifugation at 12,000 rpm for 30 min at 4°C. Proteins were quantified using BCA method and separated with 10% SDS-PAGE. Proteins were transferred onto a polyvinylidene difluoride (PVDF) membrane, and then incubated in 1× TBST containing 5% milk for 2 h at room temperature. The PVDF membrane was incubated with anti-human primary antibodies for overnight at 4°C, and then with anti-rabbit secondary antibody at room temperature for 1.5 h. According to the manufacturer’s instructions, the gel electrophoresis image analyzer GDS8000 (Thermo Fisher Scientific) was then used to detect signals with ECL reagent. The relative protein expression was analyzed by Image-J software, represented as the density ratio versus GAPDH.

### Construction of Lentiviral Vector and Preparation of Recombinant Lentivirus

Lentiviral CRISPR/cas9 mediated PHGDH gene editing vector was constructed by annealing gRNA oligonucleotide pairs and subcloning them into pLenti-Cas9-sgRNA-puro lentivirus vector (Genechem Co., Ltd.). The gene encoding PHGDH labeled with Myc epitope (Myc-PHGDH) and RMRP were cloned into pGCLV-GV166 lentivirus vector (Genechem Co., Ltd.). The shRNA targeting RMRP was designed and cloned into GV118 lentivirus vector (Genechem Co., Ltd.). The RNA sequence information was shown in the **Supplementary Table 1**. Genechem Co., Ltd. performed DNA sequencing to verify the sequence of the insert and identified it as 100%. After construction, the recombinant lentiviral vector, plasmid pHelper 1.0 and plasmid pHelper 2.0 were co-transfected into 293T cells with liposome 3000 (Invitrogen). The recombinant lentivirus was harvested 72 h after transfection, centrifuged to remove cell debris, and then filtered through 0.22 μm cellulose acetate filter. The final titer of lentivirus was 1.0 × 10^9^ Tu/ml.

### Gene Expression Mediated by Recombinant Lentivirus

Cells were infected with recombinant virus targeting genes and corresponding empty vector without gene targeting as negative control for 12 h. After 72 h, the infected cells were subjected to puromycin selection. Western blot was used to measure the infection efficiency of each gene and the negative control. Specially, to purify the PHGDH in cells, cells were infected recombinant virus expressing PHGDH tagged with Myc epitope (Myc-PHGDH) for 12 h. After 72 h, the infected cells were subjected to puromycin selection. Western blot was used to measure the infection efficiency of each gene and the negative control, where the PHGDH expression level of Myc-PHGDH and the control group was detected by incubating Myc-antibody as the primary antibody. Then cells were treated as indicated and subjected to further analysis.

### 3-(4,5-Dimethylthiazol-2-yl)-2,5-Diphenyltetrazolium Bromide (MTT) Assay

The MTT assay was performed according to the manufacturer’s instruction. Briefly, cells were exposed to different concentrations of cisplatin for 48 h, cells were incubated with MTT for 4 h, followed by addition of isopropyl alcohol to dissolve the formazan crystals. OD was measured at 570 nm using a Microplate Reader.

### Generation of Reporter Vectors and Dual-Luciferase Reporter Assay

The 5′UTR (untranslational region), CDS (coding sequence), and 3′UTR fragments of PHGDH mRNA was generated by PCR and inserted into the pMIR-REPORTTM Luciferase vector (Promega, Madison, WI) just after the stop codon. The transfection was carried out with Lipofectamine 3000 (Invitrogen) according to the manufacturer’s instructions. Cells were incubated for 48 h and harvested by adding 100 µl of reporter lysis buffer (Dual-Luciferase Assay System, Promega). Luciferase activity was measured by Dual Luciferase Reporter Gene Assay Kit. Experiments were performed in triplicates and repeated for three times independently. The Renilla luciferase activity values that reflect transfection efficiency were utilized to normalize the firefly luciferase activity values. Data are presented as mean values (s.d.).

### Transwell Migration and Matrigel Invasion Assays

Transwell invasion and migration assays were performed to determine cell invasion and migration, respectively. Transwell inserts coated with Matrigel on the upper layers were used for invasion assay. Uncoated inserts were used for migration assay. Briefly, cells were seeded into the upper chamber with FBS-free medium, and lower chamber was filled with full medium. The cells were incubated in a humidified 5% CO_2_ incubator at 37°C for 24h. The invaded or migrated cells were fixed in 4% paraformaldehyde for 5min and then stained with 0.3% crystal violet. Invading cells or migrating cells were counted under a light microscope.

### Polysome Profiling

Some 1 × 10^7^ cells were incubated with 100 μg/ml cycloheximide for 5 min to halt elongation. Cells were then harvested in 500 μl of polysome lysis buffer (15 mM Tris–HCl pH 7.4, 15 mM MgCl_2_, 0.3 M NaCl, 1 mg/ml heparin, 0.1 mg/ml cycloheximide, and 1% Triton X-100) and centrifuged at 13,000 rpm for 10 min at 4°C. The supernatants were then loaded on a sucrose density gradient system ranging from 7 to 47% (Teledyne Isco). The gradients were centrifuged in an SW41 Beckman rotor at 35,000 rpm for 180 min at 4°C. Gradients were collected into 0.5 ml/tube fractions by monitoring RNA absorbance at 254 nm using an ISCO fractionator (Brandel, Inc.). RNA was isolated from monosome, and polysome fractions using the TRIzol reagent (Life Technologies) and quantified. Reverse transcription (RT) reactions were performed using a SuperScript III first-strand synthesis system (Life Technologies) with a random primer. All polysomal analysis was done a minimum of three times.

### Clinical Samples

In the present study, 25 patients were recruited in this study, who underwent surgical resection at Shengjing Hospital of China Medical University from June 2014 to July 2017. The collected tissues were immediately frozen in liquid nitrogen and stored in −80°C freezer till further utilization. The patients were divided into two groups, platinum-sensitive (sixteen patients) and platinum resistant (nine patients) groups with ages of 43 to 69 years (57 on the average). None of the patients had received chemotherapy or radiotherapy prior to the operation. The project was approved by Institutional Review Board of China Medical University that informed consent was not needed to obtain from the patients or their family.

### Tissue Microarray and Immunohistochemical Staining

Tissue microarray sections were purchased from Shanghai Outdo Biotech Co., LTD. Immunohistochemical staining was performed on tissue sections using an antibody against PHGDH. A semi-quantitative H-score was assessed for each specimen by multiplying the distribution areas (0–100%) at each staining intensity level by the intensities (0: negative; 1: weak staining; 2: moderate staining; 3: strong staining) as previously reported (30). The median value of the H-score was chosen as the cutoff criterion to categorize into high and low expression subgroup.

### Statistical Analysis

The statistical significance of the difference was analyzed by analysis of variance (ANOVA) and *post hoc* Dunnett’s test. Statistical significance was defined as *P <* 0.05. All experiments were repeated three times, and data were expressed as the mean ± SD (standard deviation) from a representative experiment.

## Results

### PHGDH Is Upregulated in Platin-Resistant Ovarian Cancer Cells and Predicts Poor Prognosis

**Figure 1:**
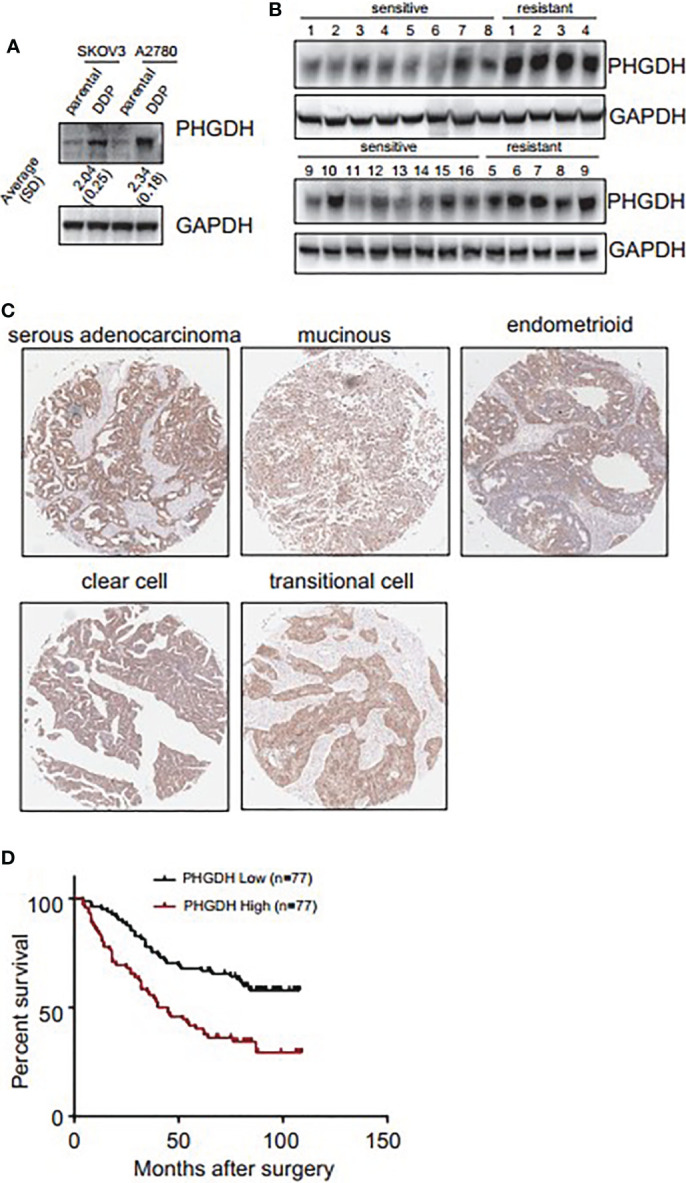
PHGDH is increased in platin-resistant ovarian cancer and predicts poor prognosis of patients with ovarian cancer. **(A)** PHGDH expression was assessed using Western blot in platin-sensitive (parental) and their platin-resistant (DDP) SKOV3 and A2780 cells. The relative expression was noted under the blots. **(B)** PHGDH expression was investigated in 16 cisplatin-sensitive and nine cisplatin-resistant ovarian cancer tissues using Western blot. **(C)** Ovarian cancer tissue microarray was subjected to immunohistochemical staining, and representative images of immunohistochemistry staining with PHGDH were presented. **(D)** Kalpan–Meier plot shows the overall survival of patients with ovarian cancer grouped by PHGDH expression.

Western blot demonstrated that PHGDH was increased in platin-resistant (DDP) SKOV3 and A2780 cells, when compared with their relative platin-sensitive parental partners (**Figure 1A**). PHGDH expression was also further investigated in 16 platin-sensitive and eight platin-resistant ovarian cancer tissues. PHGDH expression was higher in most of platin-resistant ovarian cancer tissues than in platin-sensitive tissues (**Figure 1B**). PHGDH expression was then investigated in a panel of ovarian cancer tissues using immunohistochemical staining. PHGDH was expressed in 118 of 154 epithelial ovarian cancer specimens (**Figure 1C**). Based on the expression of PHGDH expression, patients with ovarian cancer were grouped into high and low expression groups. Kaplan–Meier survival analysis demonstrated that high PHGDH predicted significantly poor overall survival of patients with ovarian cancer (**Figure 1D**).

### Knockdown of PHGDH Increases Responsiveness to Cisplatin and Suppresses Capacities of Invasion and Spheroid Formation in Platin-Resistant Ovarian Cancer

**Figure 2:**
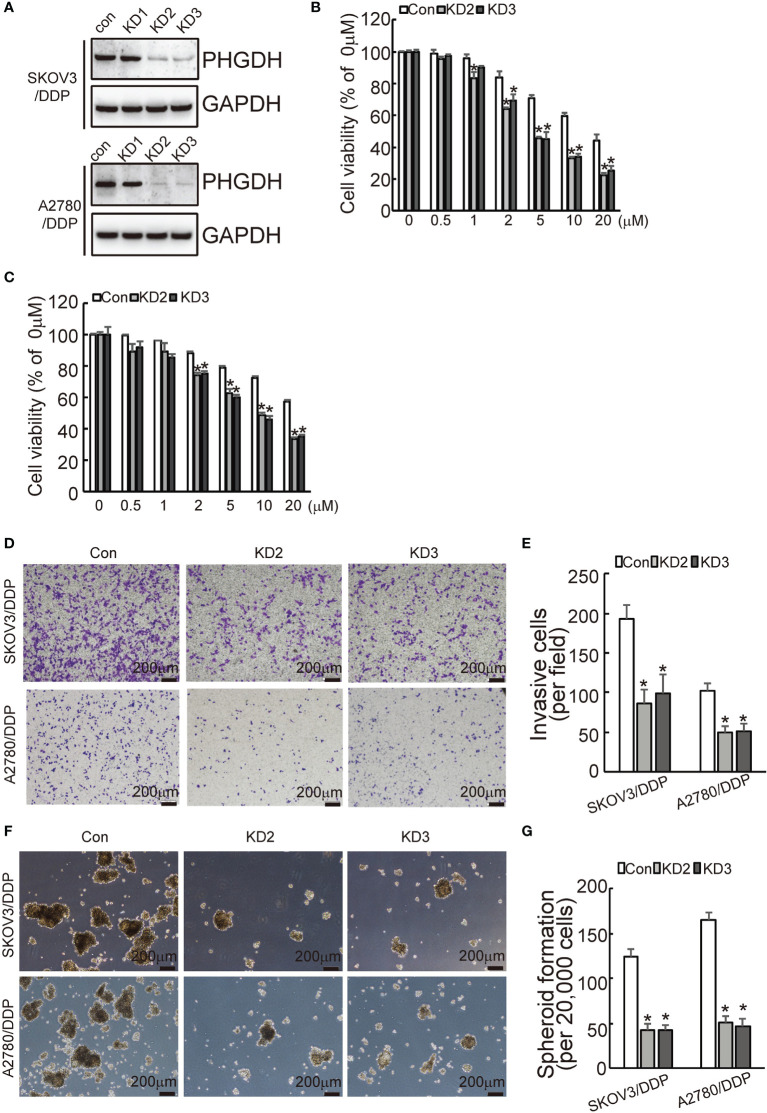
PHGDH knockdown increases responsiveness to cisplatin and decreases capacities of invasion and spheroid formation in platin-resistant ovarian cancer. **(A)** SKOV3/DDP or A2780/DDP cells were infected with CASPR-Cas9 lentivirus containing specific gRNA against PHGDH and the pLenti-Cas9-sgRNA-puro lentivirus vector without PHGDH targeting as control, knockdown of PHGDH was confirmed by Western blot. **(B, C)** The indicated cells were treated with the solvent control and different doses of cisplatin for 48 h, and cell viability was analyzed using CCK8 assays. **(D, E)** The indicated cells were plated on the Matrigel-coated transwell, invaded cells were stained with crystal violet and photographed **(D)**, cell numbers were counted and plotted **(E)**. **(F, G)** The indicated cells were floating cultured with serum-free media for 14 days, spheroid was photographed **(F)**, and spheroid numbers were counted and plotted **(G)**. *P < 0.01; N.S., not significant.

To investigate the potential role of PHGDH in ovarian cancer, PHGDH was knocked down using CASPR-Cas9 system. Tow of gRNAs against PHGDH significantly decreased PHGDH expression in SKOV3/DDP and A2780/DDP cells compared to the cells infected with the pLenti-Cas9-sgRNA-puro lentivirus vector without targeting PHGDH (**Figure 2A**). CCK8 assays demonstrated the knockdown of PHGDH significantly decreased cell viability of SKVO3/DDP (**Figure 2B**) and A2780/DDP (**Figure 2C**) cells when exposed to cisplatin. Matrigel-coated Transwell assay demonstrated that knockdown of PHGDH significantly decreased invasiveness of SKOV3/DDP and A2780/DDP cells (**Figures 2D, E**). In addition, PHGDH knockdown also suppressed spheroid formation capacity of SKOV3/DDP and A2780/DDP cells (**Figures 2F, G**).

### Ectopic Expression of PHGDH Decreases Responsiveness to Cisplatin and Promotes Invasiveness and Spheroid Formation of Ovarian Cancer Cells

**Figure 3:**
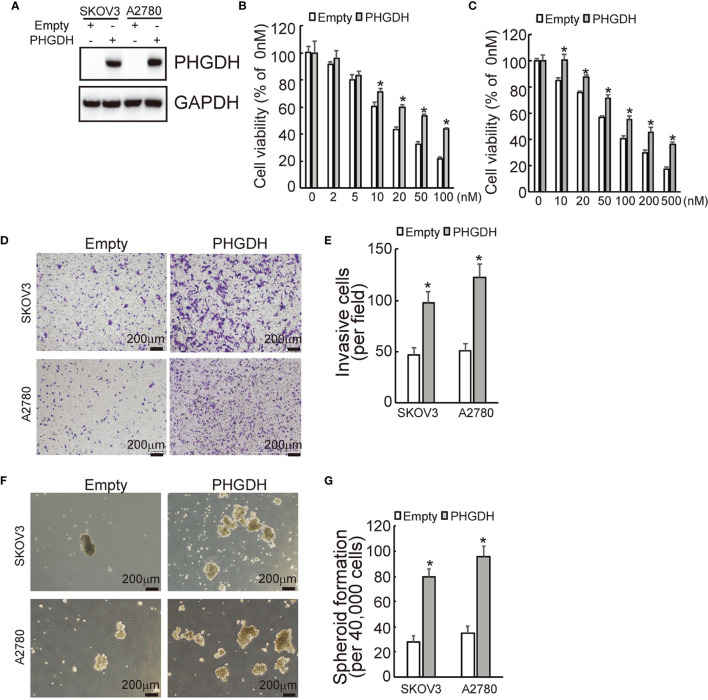
PHGDH overexpression suppresses responsiveness to cisplatin and promotes invasion and spheroid formation of platin-sensitive ovarian cancer cells. **(A)** SKOV3 or A2780 cells were infected with lentivirus containing PHGDH labeled with Myc epitope (Myc-PHGDH, labeled as PHGDH) and the empty pGCLV-GV166 lentivirus vector (empty) as control, expression of PHGDH was confirmed by Western blot. **(B, C)** The indicated cells (SKOV3 or A2780 cells were infected with lentivirus containing PHGDH (PHGDH) and the empty pGCLV-GV166 lentivirus vector (empty) as control) were treated with the solvent control and were treated with different doses of cisplatin for 48 h, and cell viability was analyzed using CCK8 assays. **(D, E)** The indicated cells were plated on the Matrigel-coated tranwell, invaded cells were stained with crystal violet and photographed **(D)**, cell numbers were counted and plotted **(E)**. **(F, G)** The indicated cells were floating cultured with serum-free media for 14 days, spheroid was photographed **(F)**, and spheroid numbers were counted and plotted **(G)**. *P < 0.01; N.S., not significant.

To further confirmed the potential role of PHGDH in ovarian cancer, PHGDH was ectopically overexpressed in SKOV3 and A2780 cells infected with lentivirus containing PHGDH labeled with Myc epitope (Myc-PHGDH) compared to SKOV3 and A2780 cells infected with the empty pGCLV-GV166 lentivirus vector (empty) (**Figure 3A**). In addition, SKOV3 or A2780 cells were infected with lentivirus containing PHGDH (PHGDH) and the empty pGCLV-GV166 lentivirus vector (empty) as control. Overexpression of PHGDH significantly increased cell viability of SKOV3 (**Figure 3B**) and A2780 (**Figure 3C**) cells upon cisplatin treatment. Matrigel-coated transwell assays demonstrated that overexpression increased invasion capacity of SKOV3 and A2780 cells (**Figures 3D, E**). In addition, PHGDH overexpression also increased spheroid formation of SKOV3 and A2780 cells (**Figures 3F, G**).

### PHGDH Is Increased in Cisplatin-Resistant Ovarian Cancer Cells at the Translational Initiation Level

**Figure 4:**
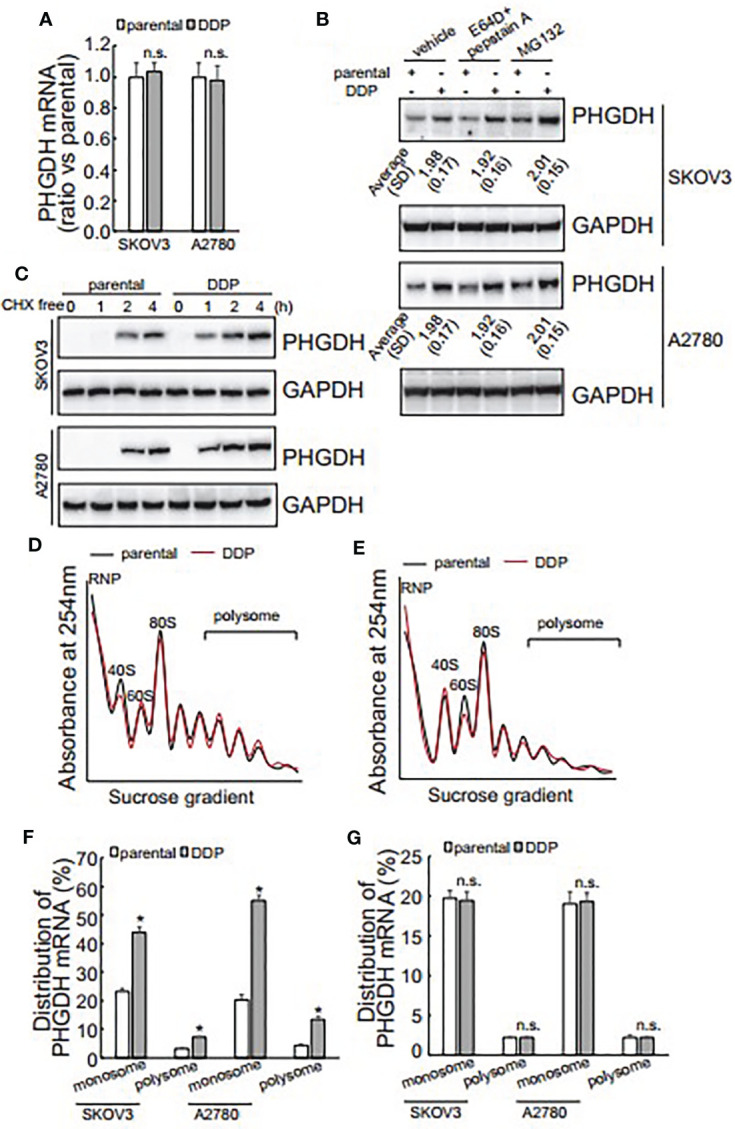
Translational activation is responsible for upregulation of PHGDH in platin-resistant ovarian cancer cells. **(A)** PHGDH mRNA expression was analyzed using real-time RT-PCR in ovarian cancer cell lines. **(B)** PHGDH expression was analyzed using Western blot after cells were incubated with the solvent vehicle, MG132, or E64D and pepstatin A for 24 h. **(C)** Ovarian cancer cells were treated with cycloheximide (CHX) for 16 h, then CHX were completely washed out. PHGDH expression was analyzed using Western blot analysis after Cells were cultured under complete media for the additional indicated time. **(D, E)** SKOV3 **(D)** or A2780 **(E)** Cells were exposed to CHX for 10 h, cell homogenate was subjected for sucrose gradient fractionation. Absorbance at 254 nm was measured in each fraction. **(F, G)** total RNA was isolated from monosome and polysome fractions, PHGDH mRNA **(F)** and GAPDH mRNA **(G)** occupation on monosome and polysome fractions was analyzed using real-time RT-PCR. *P < 0.05, n.s., not significant.

The promotive role of PHGDH in cisplatin-resistant, invasion and spheroid formation of ovarian cancer cells promoted us to further investigate the underlying mechanisms of its upregulation in platin-resistant ovarian cancer. qRT-PCR found that no obvious alteration of PHGDH mRNA was observed in platin-resistant SKOV3 and A2780 cells when compared with their platin-sensitive partners (**Figure 4A**), indicating that PHGDH was upregulated in platin-resistant ovarian cancer cells at the protein level. E64D plus pepstatin A and MG132 were then utilized to suppress lysosomal and proteasomal degradation of PHGDH, respectively. Neither E64D plus pepstatin A nor MG132 altered the different expression of PHGDH in platin-sensitive ovarian cancer cells and their platin-resistant partners compared to the cells treated with the solvent vehicle (**Figure 4B**), indicating that synthesis, but not degradation of PHGDH might be responsible for upregulation of PHGDH in platin-resistant ovarian cancer cells. Cells were then treated with Cycloheximide (CHX) for 16 h to completely block protein synthesis, then CHX was washed out to resume protein synthesis for different period. PHGDH expression was apparently recovered after 1 h free of CHX in SKOV3/DDP and A2780/DDP cells, while its expression was delayed in their parental partners (**Figure 4C**), confirming that PHGDH translation is activated in platin-resistant ovarian cancer cells. PHGDH mRNA distribution was also investigated using ribosome profiling. Distribution of total RNAs was similar between platin-resistant and platin-sensitive SKOV3 (**Figure 4D**) and A2780 (**Figure 4E**) cells. Occupation of PHGDH mRNA on both monosomes and polysomes was significantly augmented in SKOV3/DDP and A2780/DDP cells when compared with their parental control partners (**Figure 4F**). On the other hand, distribution of GAPDH was not different between platin-resistant ovarian cancer cells and their parental control partners (**Figure 4G**).

### DDX3X Recruitment on PHGDH mRNA Is Increased and Implicated in Translation of PHGDH in Platin-Resistant Ovarian Cancer Cells

**Figure 5:**
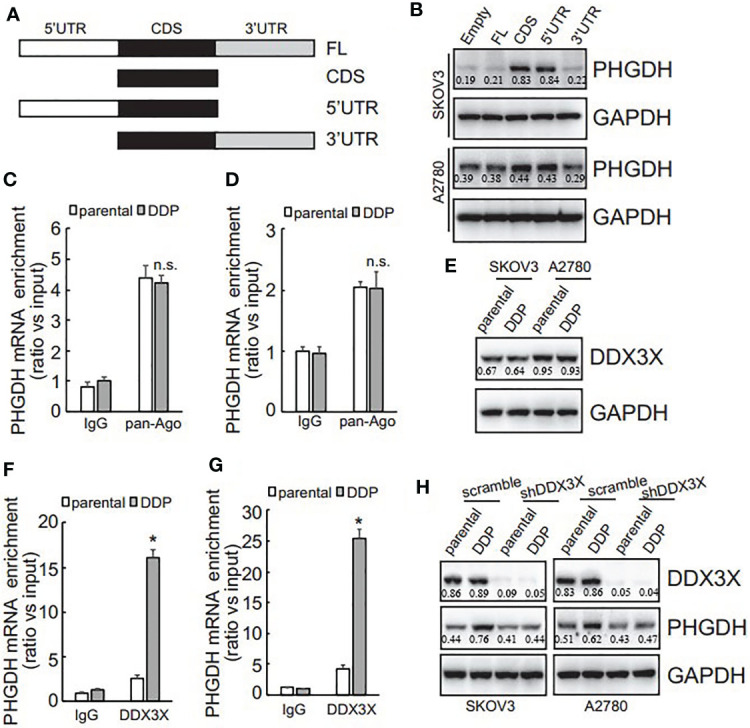
PHGDH expression is upregulated *via* 3’UTR of its transcript in a DDX3X-dependent pattern. **(A)** The PHGDH expression pMIR-REPORTTM Luciferase vectors were cloned as illustrated. **(B)** cells were transfected with empty pMIR-REPORTTM Luciferase vector (empty) and PHGDH vector (FL, CDS, 5’UTR, 3’UTR), and ectopic PHGDH expression was investigated using Western blot analysis. **(C, D)** RIP was performed using IgG or pan-Ago antibody from control and DDP paired A2780 **(C)** or SKOV3 **(D)** cells, and enrichment of PHGDH mRNA was evaluated using qRT-PCR. **(E)** DDX3X expression was analyzed using Western blot analysis. **(F, G)** RIP was performed using IgG or DDX3X antibody from control and DDP paired A2780 **(F)** or SKOV3 **(G)** cells, and enrichment of PHGDH mRNA was evaluated using qRT-PCR. **(H)** Cells were infected with pGCLV-GV166 lentivirus vector (scramble) or shRNAs against DDX3X (shDDX3X), PHGDH expression was analyzed using Western blot. *P < 0.05, n.s., not significant.

To investigate the potential responsive cis-acting element on PHGDH transcript, eukaryotic expression constructs containing various fragment of PHGDH transcript were generated as illustrated (**Figure 5A**). Transfection of eukaryotic expression construct containing CDS alone or 5’UTR and CDS significantly increased PHGDH expression, while constructs containing 3’UTR fragment was not overexpressed efficiently in SKOV3 and A2780 cells (**Figure 5B**), indicating that 3’UTR of PHGDH might suppress its translation. miRNAs containing RISC are well-known to regulate translation *via* 3’UTR of target mRNAs, RNA immunoprecipitation (RIP) was then performed using pan-Ago antibody, a key component of RISC. Enrichment of PHGDH mRNA by pan-Ago was not significantly different between platin-resistant and platin-sensitive SKOV3 (**Figure 5C**) and A2780 (**Figure 5D**) cells, indicating that miRISCs might not be implicated in differential translation of PHGDH between platin-resistant and platin-sensitive ovarian cancer cells. In addition to miRNAs, RNA-binding proteins are also involved in translation of target mRNAs. Using biotin-labeled 3’UTR of PHGDH transcript, quantitative analysis of PHGDH RNA binding proteins were evaluated by biotin pull down followed by quantitative mass spectrometry. Among differentially potential PHGDH mRNA-binding proteins, recruitment of Dead-box helicase 3 X-linked (DDX3X) was significantly increased. Western blot showed that no apparent difference of DDX3X expression was observed in platin-resistant and platin-sensitive SKOV3 and A2780 cells (**Figure 5E**), while DDX3X recruitment on PHGDH mRNA was significantly increased in platin-resistant SKOV3 (**Figure 5F**) and A2780 (**Figure 5G**) cells, when compared with their parental control partners. In addition, knockdown of DDX3X significantly decreased PHGDH expression in SKOV3/DDP and A2780/DDP cells (**Figure 5H**).

### LncRNA RMRP Is Increased and Promotes Recruitment of DDX3X on the PHGDH mRNA in Platin-Resistant Ovarian Cancer Cells

**Figure 6:**
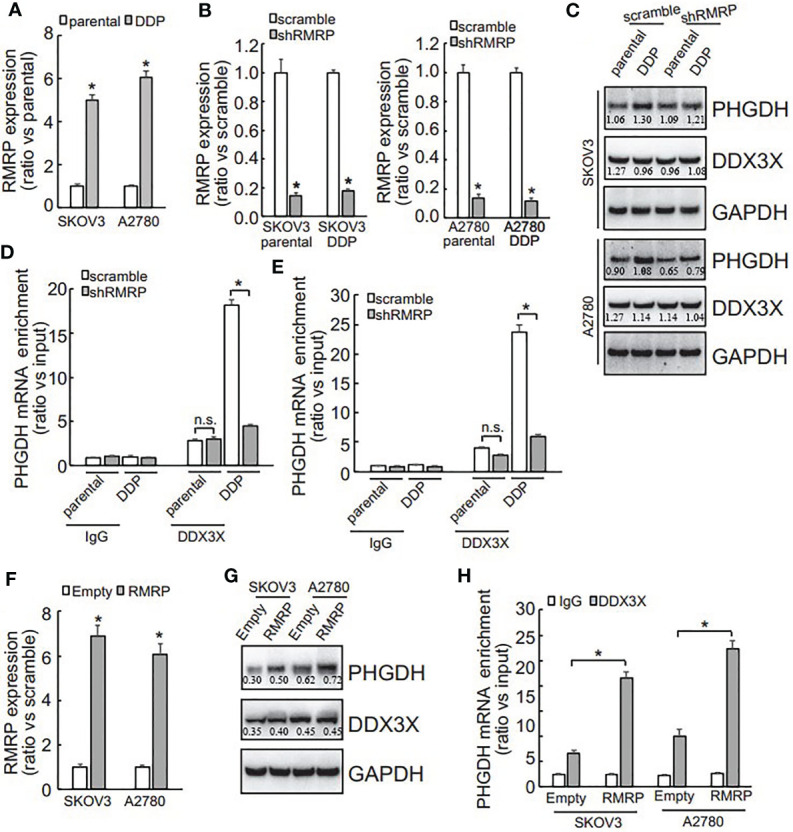
RMRP is increased and promotes recruitment of DDX3X to PHGDH transcript in cisplatin-resistant ovarian cancer cells. **(A)** qRT-PCR was performed to analyze the RMRP expression. **(B, C)** cells were infected with scramble GV118 lentivirus vector (scramble) or shRNAs against RMRP (shRMRP), knockdown efficiency was confirmed by qRT-PCR **(B)**, PHGDH expression was analyzed using Western blot **(C)**. **(D, E)** RIP was performed using IgG or DDX3X antibody from parental and DDP paired SKOV3 **(D)** and A2780 **(E)** infected with scramble or shRMRP. **(F, H)** cisplatin-sensitive SKOV3 and A2780 cells were infected with empty pGCLV-GV166 lentivirus vector (empty) or lentivirus containing RMRP (RMRP), or RMRP, expression of RMRP was analyzed using qRT-PCR **(F)**, PHGDH expression was investigated using Western blot **(G)**, recruitment of DDX3X on PHGDH transcript was studied using RIP **(H)**. *P < 0.05, n.s., not significant.

DDX3X expression was not different (**Figure 5E**), while its enrichment on the PHGDH mRNA was increased (**Figures 5F, G**), indicating that other factor(s) might guide DDX3X to the PHGDH mRNA in platin-resistant ovarian cancer. Since except for RNA binding proteins, non-coding RNAs are also implicated in translation of target mRNAs *via* interaction, non-coding RNAs that can potentially interact with both PHGDH mRNA and DDX3X were screened *via* online database (starbase.sysu.edu.cn) and RNA component of mitochondrial RNA processing endoribonuclease (RMRP) attracted our attention. qRT-PCR demonstrated that RMRP was significantly increased in SKOV3/DDP and A2780/DDP cells (**Figure 6A**), while LINC00662 was undetectable in any samples. RMRP was then knocked down in SKOV3 (**Figure 6B**) and A2780 (**Figure 6C**) cells. Western blot demonstrated that knockdown of RMRP significantly decreased PHGDH expression in SKOV3/DDP and A2780/DDP cells (**Figure 6**). RIP was also performed and demonstrated that recruitment of DDX3X on the PHGDH mRNA was significantly compromised by RMRP knockdown in SKOV3/DDP (**Figure 6D**) and A2780/DDP (**Figure 6E**) cells. RMRP was also ectopically expressed in platin-sensitive SKOV3 and A2780 cells (**Figure 6F**). Overexpression of RMRP significantly increased PHGDH expression in SKOV3 and A2780 cells (**Figure 6G**). In addition, overexpression of RMRP also promoted recruitment of DDX3X on the PHGDH mRNA in SKOV3 and A2780 cells (**Figure 6H**).

## Discussion

Ovarian cancer is rarely diagnosed during its early stages since lack of obvious symptoms, which ranks the fifth leading cause of cancer-related death in female. Standard treatment for ovarian cancer includes staging and optimal debulking surgery followed by adjuvant platinum-based (*e.g.* cisplatin-based) chemotherapy as the first line treatment. Despite the apparent benefits for patients with ovarian cancer utilizing platinum-based chemotherapy, initial or acquired resistance is a clinical barrier leading to therapeutic failure. Therefore, it is of clinical significance to better understand the mechanisms underlying cisplatin resistance. Metabolic reprogramming is one of the characteristics of tumor cells, which is closely related to tumorigenesis, progression and drug resistance. As for intracellular glucose metabolism pathway, in addition to glycolysis and mitochondrial oxidative phosphorylation, there are also some branch pathways, such as serine synthesis pathway. The expression of PHGDH, the rate limiting enzyme of serine metabolism pathway, increased significantly in cisplatin resistant cells, suggesting that serine metabolism rearrangement occurred in cisplatin resistant cells. Serine is essential for cell synthesis of nucleotides, proteins and lipids. Many types of cancer need to synthesize serine to maintain a rapid and stable growth pattern. PHGDH is a key enzyme in serine biosynthesis pathway, which plays a key role in serine biosynthesis and mitochondrial redox homeostasis in cancer cells. Furthermore, we found that PHGDH knockout inhibited the proliferation of ovarian cancer cells, while PHGDH overexpression increased the survival rate, invasiveness and spheroid formation of ovarian cancer cells after cisplatin exposure. The current study demonstrated that PHGDH, the key enzyme implicated in serine biosynthesis pathway, was upregulated in cisplatin-resistant ovarian cancer cells and tissues. Knockdown of PHGDH decreased cell viability upon cisplatin exposure, suppressed invasion and spheroid formation of cisplatin-resistant ovarian cancer cells, while overexpression of PHGDH increased cell viability upon cisplatin exposure, promoted invasion and spheroid formation of cisplatin-sensitive ovarian cancer cells. Importantly, high PHGDH expression predicted a poor prognosis of patients with ovarian cancers. These data indicated that upregulation of PHGDH might play a role on cisplatin resistance, and PHGDH might function as an oncogene in ovarian cancer. Therefore, targeting PHGDH might render resistant cells sensitive to cisplatin, providing a potential strategy for treatment of cisplatin resistant ovarian cancer.

PHGDH has been shown to be regulated at multiple levels, including gene amplification (25, 31), transcriptional activation (27), as well proteasomal degradation (29). The current study provides an additional regulatory mechanism by which PHGDH is regulated at the translational level. Our data suggest that PHGDH translational efficiency is different in platin-resistant and platin-sensitive ovarian cancer cells, which further adds diversity to the regulation of PHGDH expression in the distinct cancers. PHGDH is highly expressed in many cancer cells and plays a critical role to support a variety biosynthetic processes important for cell proliferation) (14, 32, 33), thereby our findings might provide a novel potential therapeutic targets for cancer therapy.

DDX3X is a member of the Asp-Glu-Ala-Asp (DEAD) box protein family. DEAD motif containing proteins have ATP-dependent RNA helicase activity, which are involved in alteration of RNA secondary structure, thereby implicated in a variety of cellular processes such as splicing and translation initiation. Nuclear DDX3X is involved in transcriptional regulation, mRNP assembly, pre-mRNA splicing, and mRNA export, while cytoplasmic DDX3X is thought to be implicated in translation, cellular signaling, and viral replication (34–36). Dysregulation of DDX3X has been implicated in tumorigenesis and development (37–44). The current study found that although total DDX3X expression was not distinct between cisplatin-sensitive and cisplatin-resistant ovarian cancer cells, while its recruitment on PHGDH mRNA was augmented in cisplatin-resistant ovarian cancer cells. Importantly, knockdown of DDX3X significantly decreased PHGDH expression in cisplatin-resistant ovarian cancer cells. These data indicated that DDX3X enrichment might play a critical role in promoting PHGDH translation in cisplatin-resistant ovarian cancer cells, while additional factor(s) are necessary for its recruitment to the PHGDH mRNA. The current study found that long non-coding RNA (Lnc RNA) RPRM was upregulated in cisplatin-resistant ovarian cancer cells and promoted enrichment of DDX3X on the PHGDH mRNA. Thereby, our findings identified cooperation of DDX3X and RPRM to promote translation of PHGDH transcript in cisplatin-resistant ovarian cancer cells.

In conclusion, the current study demonstrates that PHGDH is upregulated and plays a critical role in cisplatin resistance in ovarian cancer cells. We show that high PHGDH expression is correlated with lower overall survival of patients with ovarian cancer. We show that PHGDH is upregulated at the translational level in cisplatin resistant ovarian cancer cells, which is regulated by recruitment of DDX3X on the PHGDH transcript. In addition, the current study also demonstrates that LncRNA RPRM is upregulated, which plays an important role in promotion of DDX3X recruitment. Thus, this study suggests that targeting PHGDH may provide a potential opportunity to overcome cisplatin resistance in ovarian cancer.

## Conclusions

Collectively, the current study described that PHGDH was upregulated and conferred resistance of ovarian cancer cells to cisplatin, suggesting that cisplatin resistance could be overcome by targeting PHGDH. In addition, our study also provided evidence that differential PHGDH protein expression was defined by its translation, and RNA binding protein DDX3X and LncRNA RMRP are regulators of its translation.

## Data Availability Statement

The original contributions presented in the study are included in the article/**Supplementary Material**. Further inquiries can be directed to the corresponding author.

## Author Contributions

QY designed the article. FB and TS did the experiments. YA and YY wrote the manuscript. All the figures were prepared by FB and revised by QY. All authors contributed to the article and approved the submitted version.

## Funding

Supported by grants from National Natural Science Foundation of China (No.81872125), 345 Talent Project of Shengjing Hospital of China Medical University (No.M0695), and Outstanding Scientific Fund of Shengjing Hospital (No. 201704).

## Conflict of Interest

The authors declare that the research was conducted in the absence of any commercial or financial relationships that could be construed as a potential conflict of interest.
