# Corrigendum: PHGDH Is Upregulated at Translational Level and Implicated in Platin-Resistant in Ovarian Cancer Cells

**DOI:** 10.3389/fonc.2022.891191

**Published:** 2022-04-25

**Authors:** Fangfang Bi, Yuanyuan An, Tianshui Sun, Yue You, Qing Yang

**Affiliations:** Department of Obstetrics and Gynecology, Shengjing Hospital of China Medical University, Shenyang, China

**Keywords:** PHGDH, DDX3X, platin-resistant, ovarian cancer cells, RMRP

In the original article, there was a mistake in **Figures 2** and **3** as published. In **Figure 2D**, we put the wrong picture of transwell result in A2780/DDP group. In **Figure 3D**, we put the wrong picture of transwell result in A2780 group. The corrected **Figures 2** and **3** appear below.

**Figure 2:**
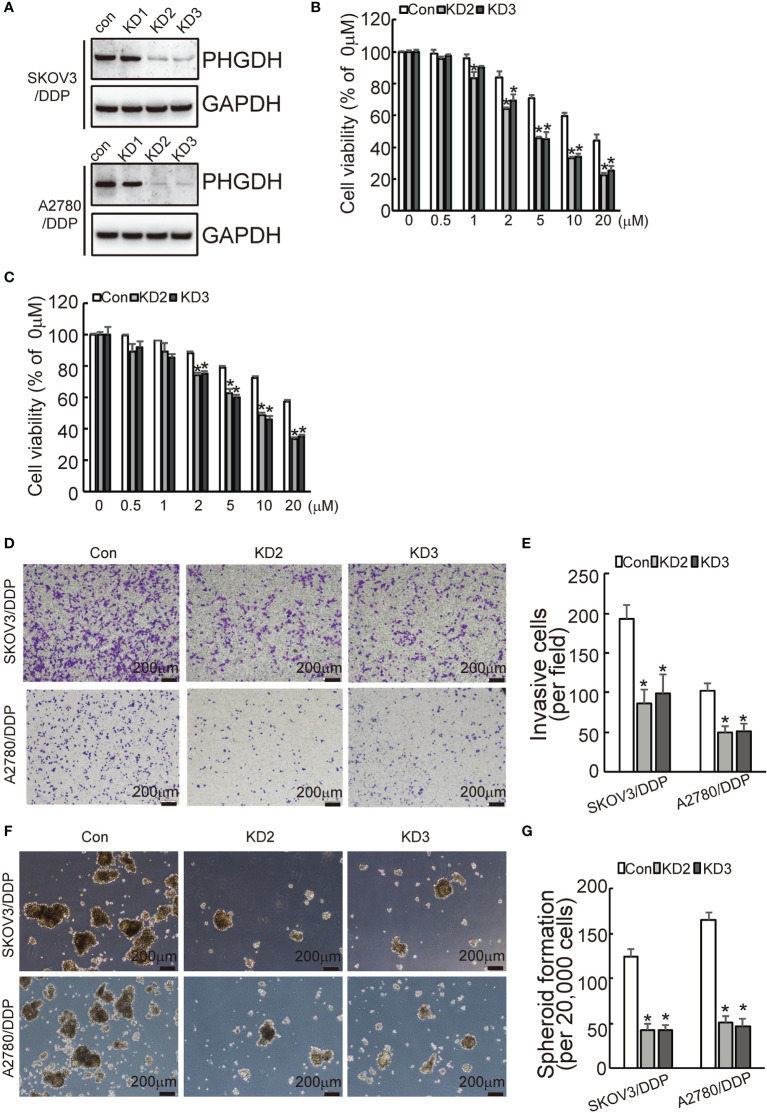
PHGDH knockdown increases responsiveness to cisplatin and decreases capacities of invasion and spheroid formation in platin-resistant ovarian cancer. **(A)** SKOV3/DDP or A2780/DDP cells were infected with CASPR-Cas9 lentivirus containing specific gRNA against PHGDH and the pLenti-Cas9-sgRNA-puro lentivirus vector without PHGDH targeting as control, knockdown of PHGDH was confirmed by Western blot. **(B, C)** The indicated cells were treated with the solvent control and different doses of cisplatin for 48 h, and cell viability was analyzed using CCK8 assays. **(D, E)** The indicated cells were plated on the Matrigel-coated transwell, invaded cells were stained with crystal violet and photographed **(D)**, cell numbers were counted and plotted **(E)**. **(F, G)** The indicated cells were floating cultured with serum-free media for 14 days, spheroid was photographed **(F)**, and spheroid numbers were counted and plotted **(G)**. *P < 0.01; N.S., not significant.

**Figure 3:**
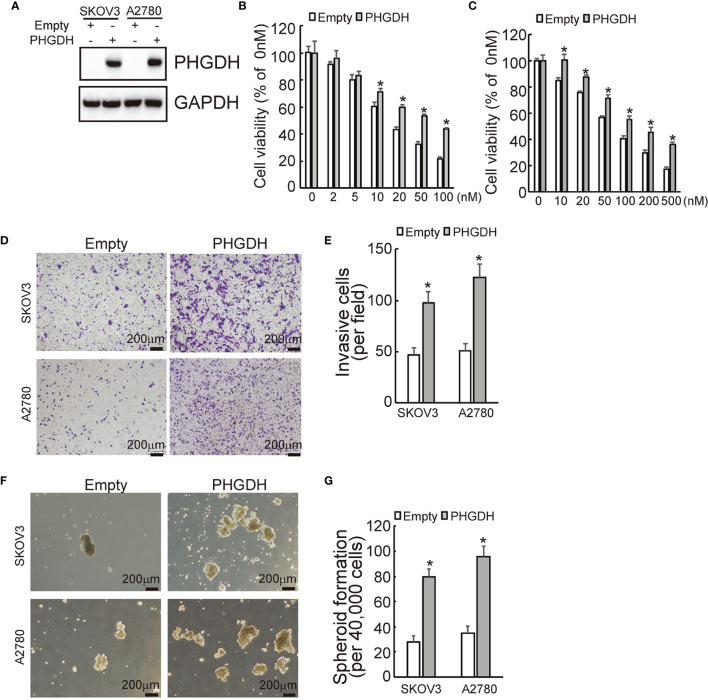
PHGDH overexpression suppresses responsiveness to cisplatin and promotes invasion and spheroid formation of platin-sensitive ovarian cancer cells. **(A)** SKOV3 or A2780 cells were infected with lentivirus containing PHGDH labeled with Myc epitope (Myc-PHGDH, labeled as PHGDH) and the empty pGCLVGV166 lentivirus vector (empty) as control, expression of PHGDH was confirmed by Western blot. **(B, C)** The indicated cells (SKOV3 or A2780 cells were infected with lentivirus containing PHGDH (PHGDH) and the empty pGCLV-GV166 lentivirus vector (empty) as control) were treated with the solvent control and were treated with different doses of cisplatin for 48 h, and cell viability was analyzed using CCK8 assays. **(D, E)** The indicated cells were plated on the Matrigel-coated tranwell, invaded cells were stained with crystal violet and photographed **(D)**, cell numbers were counted and plotted **(E)**. **(F, G)** The indicated cells were floating cultured with serum-free media for 14 days, spheroid was photographed **(F)**, and spheroid numbers were counted and plotted **(G)**. *P < 0.01; N.S., not significant.

The authors apologize for this error and state that this does not change the scientific conclusions of the article in any way. The original article has been updated.

## Publisher’s Note

All claims expressed in this article are solely those of the authors and do not necessarily represent those of their affiliated organizations, or those of the publisher, the editors and the reviewers. Any product that may be evaluated in this article, or claim that may be made by its manufacturer, is not guaranteed or endorsed by the publisher.

