# Supplementary material for: PHGDH Is Upregulated at Translational Level and Implicated in Platin-Resistant in Ovarian Cancer Cells
Source: Front Oncol. 2021 Jun 10;11:643129. doi: 10.3389/fonc.2021.643129 (PMC8222667; doi:10.3389/fonc.2021.643129)
Supplement: Supplementary file 1 [file Table_1.docx]

***Supplementary tables***

*Table 1 RNA sequences of the targeted genes*

|  | *Forward* | *Reverse* |
| --- | --- | --- |
| *PHGDH gRNA 1* | *5’-ACGGCGCAGCTGAGAAACTCCAGG-3’* | *5’-CAAAGGAGTTTCTCAGCTGCGCCGT-3’* |
| *PHGDH gRNA 2* | *5’-ACGGTATTGTTCGCTCTGCCACCA-3’* | *5’-CAAATGGTGGCAGAGCGAACAATA-3’* |
| *PHGDH gRNA 3* | *5’-ACGGAGGTGTGGACAATGTGGATC-3’* | *5’-CAATGATCCACATTGTCCACACCT-3’* |
| *PHGDH overexpression* | *5’-CAGGATCCACTCCAGCAATGGCTTTTGC-3’* | *5’- CCAAGCTTGAAGTGGAACTGGAAGGCTTC-3’* |
| *RPRM shRNA* | *5’- CACCGCTTAGGTACTTCTACTCACACG*  *AATGTGAGTAGAAGTACCTAAGC -3’* | *5’- AAAAGCTTAGGTACTTCTACTCACATTCGTGTG*  *AGTAGAAGTACCTAAGC -3’* |
| *RPRM overexpression* | *5’-ATGGATCCCCAGTTACTCTAGCGCGCAG-3’* | *5’-GCAAGCTTTTGGGGGTAGGCTCTTTATTG-3’* |

*Table 2 RNA primer sequences*

|  | *Forward* | *Reverse* |
| --- | --- | --- |
| *PHGDH* | *TCGCTCTGCCACCAAGGTGAC* | *CACAAGTGAGTTCTGCGGCAC* |
| *GAPDH* | *TCACCATCTTCCAGGAGCGAG* | *TGGTGCAGGAGGCATTGCAG* |
| *RMRP* | *CTGTATCCTAGGCTACACAC* | *CTCAGCGGGATACGCTTCTTG* |

*Table 3 Information of the patients*

|  | *Chemosensitivity* | *Stage* |
| --- | --- | --- |
| *1* | *Sensitive* | *IIIc* |
| *2* | *Sensitive* | *IIIb* |
| *3* | *Sensitive* | *Ic* |
| *4* | *Sensitive* | *Ib* |
| *5* | *Sensitive* | *IIc* |
| *6* | *Sensitive* | *Ia* |
| *7* | *Sensitive* | *IIIb* |
| *8* | *Sensitive* | *IIIc* |
| *9* | *Sensitive* | *IIb* |
| *10* | *Sensitive* | *IIIc* |
| *11* | *Sensitive* | *IIIc* |
| *12* | *Sensitive* | *IIIc* |
| *13* | *Sensitive* | *IV* |
| *14* | *Sensitive* | *Ic* |
| *15* | *Sensitive* | *IIc* |
| *16* | *Sensitive* | *IIIc* |
| *17* | *Resistant* | *IIIc* |
| *18* | *Resistant* | *IIIc* |
| *19* | *Resistant* | *IIIc* |
| *20* | *Resistant* | *IIIc* |
| *21* | *Resistant* | *IIIc* |
| *22* | *Resistant* | *IIIc* |
| *23* | *Resistant* | *IIIc* |
| *24* | *Resistant* | *IIIc* |
| *25* | *Resistant* | *IIIc* |

*Table 4 The information about the Lnc-RMRP sequence*

| *Transcript ID* | *NR_003051.3* |
| --- | --- |
| *Species* | *Homo sapiens* |
| *Gene* | *RNA component of mitochondrial RNA processing endoribonuclease (RMRP)* |
| *Sequence* | *GGTTCGTGCTGAAGGCCTGTATCCTAGGCTACACACTGAGGACTCTGTTCCTCCCCTTTCCGCCTAGGGGAAAGTCCCCGGACCTCGGGCAGAGAGTGCCACGTGCATACGCACGTAGACATTCCCCGCTTCCCACTCCAAAGTCCGCCAAGAAGCGTATCCCGCTGAGCGGCGTGGCGCGGGGGCGTCATCCGTCAGCTCCCTCTAGTTACGCAGGCAGTGCGTGTCCGCGCACCAACCACACGGGGCTCATTCTCAGCGCGGCTGTAAAAAAAAA* |
